# LaNi_(1−*x*)_FexO_3_ perovskite catalysts prepared by high-energy ball milling for efficient air cathodes in alkaline fuel cells

**DOI:** 10.1039/d5ra06763d

**Published:** 2026-01-06

**Authors:** Chontira Sangsubun, Chakkrapong Chaiburi, Supandee Maneelok

**Affiliations:** a Faculty of Science and Digital Innovation, Thaksin University Phatthalung 93210 Thailand schontira@tsu.ac.th; b Faculty of Engineering, Thaksin University Phatthalung Campus Phatthalung 93210 Thailand chakkrapong@tsu.ac.th; c Faculty of Health and Sports Science, Thaksin University Phatthalung 93210 Thailand msupandee@tsu.ac.th

## Abstract

The LaNi_(1−*x*)_Fe_*x*_O_3_ (*x* = 0, 0.2, 0.4, 0.6, 0.8, and 1) perovskite powder was prepared using a conventional mixed-oxide method through high-energy ball milling and calcination at 800 °C. The powders were then sintered at 1000 °C for 2 hours with a heating/cooling rate of 5 °C min^−1^. The LaNi_(1−*x*)_Fe_*x*_O_3_ powders were analyzed using scanning electron microscopy (SEM), energy-dispersive X-ray spectroscopy (EDS), thermogravimetric analysis (TGA), differential scanning calorimetry (DSC), X-ray diffraction (XRD), and cyclic voltammetry (CV). From the experimental results, it was found that the SEM micrograph of the LaNi_0.6_Fe_0.4_O_3_ powder shows that the calcined LaNi_0.6_Fe_0.4_O_3_ powder has a particle size ranging from approximately 30 to 400 nm. In comparison, the sintered powder at 1000 °C has an average particle size ranging from 50 to 600 nm. The LaNi_0.6_Fe_0.4_O_3_ powder exhibits a rhombohedral phase structure. The LaNi_0.6_Fe_0.4_O_3_ catalyst, when used in a 0.1 M local sorbitol solution and 0.1 M KOH, can be effectively utilized as a catalyst for electro-oxidation electrode materials in the oxygen reduction reaction.

## Abstract 2

The LaNi_(1−*x*)_Fe_*x*_O_3_ (*x* = 0, 0.2, 0.4, 0.6, 0.8, and 1) perovskite powder was prepared using a conventional mixed-oxide method through high-energy ball milling and calcination at 800 °C.

## Graphical abstract

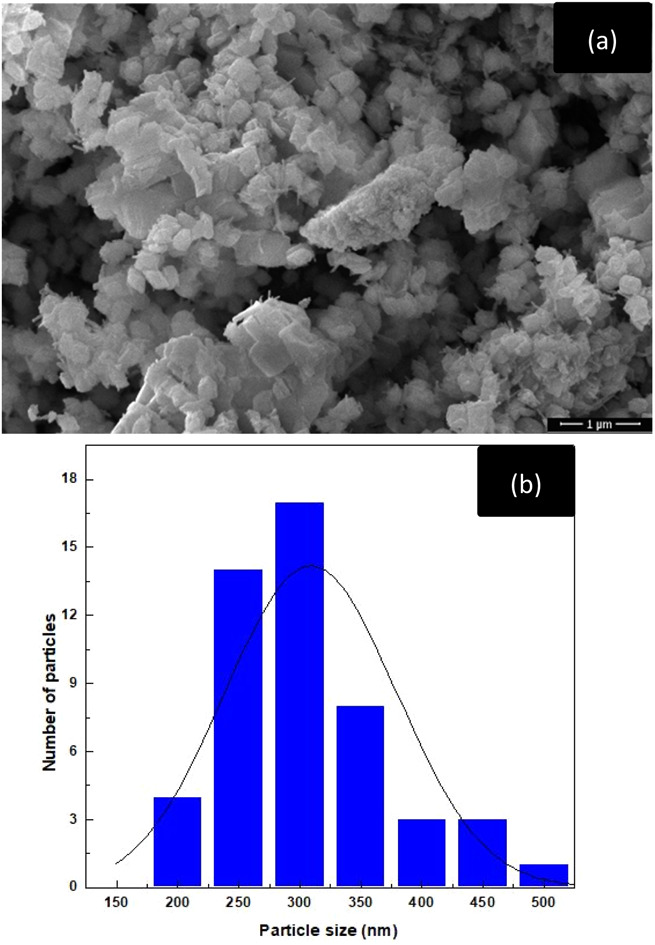

## Introduction

Industrial growth is experiencing rapid expansion, leading to a substantial increase in electricity demand. Today's electricity generation relies on fossil fuels, predominantly coal, oil, and natural gas. However, these methods of electricity generation could be more efficient and often contribute significantly to environmental pollution. Fossil fuels, being finite resources, may result in future shortages and escalating prices. This makes the development of technologies for efficient electricity generation and the exploration of alternative energy sources crucial. In this context, fuel cells emerge as a promising option for efficient and clean energy utilization, offering a responsible and sustainable solution to our energy needs. They are gaining increasing attention for continuously producing electricity by introducing reactants into the system. The reactants, typically hydrogen gas at the anode and oxygen gas at the cathode, enable a continuous process as long as the by-products are efficiently removed from the system, with water being the final by-product, which is environmentally benign. Wang and Nehrir (2007)^1^ discuss the importance of fuel cells in efficient power generation under load transients. Fuel cells comprise several essential components, including a cathode that facilitates efficient and stable electricity flow under normal operating conditions. It typically utilizes ceramic materials with a perovskite structure, as noted by Bhalla *et al.* (2000),^2^ who reviewed the significance of perovskite materials in fuel cell applications. The anode absorbs hydrogen gas and facilitates its electrochemical reaction to convert it into water while releasing electrons. Basu *et al.* (2004)^3^ studied the microstructure and conductivity of lanthanum-based perovskite materials used in anodes. The electrolyte allows the flow of ions or electrons. The interconnect, a crucial component, connects individual cell units, ensuring a smooth flow of electricity between the cells. Sealing materials ensure proper sealing and integration of the fuel cell system components, as discussed by Larminie (2003).^4^ Moreover, fuel cells require effective and efficient cathode materials. Hou *et al.* (2014)^5^ explored the performance of cobalt-free proton-blocking composite cathodes, utilizing lanthanum-based materials for solid oxide fuel cells, emphasizing the role of proton conduction. In their studies, Jin *et al.* (2018)^6^ evaluated the electrochemical performance of Fe and Mn co-doped layered perovskite cathodes, highlighting their potential as advanced cathode materials for intermediate-temperature solid oxide fuel cells. Faverge *et al.* (2023)^7^ conducted *in situ* investigations on glucose oxidation, demonstrating insights into the mechanisms of catalytic reactions in fuel cells and furthering the understanding of the electrochemical processes that can enhance efficiency. The choice of materials plays a significant role in the efficiency and stability of fuel cells. Carvalho *et al.* (1997)^8^ examined new preparation methods for lanthanum nickelate-based compounds, revealing how composition and preparation methods affect performance. Additionally, Sun *et al.* (2011)^9^ studied proton-blocking composite cathodes, demonstrating their importance in improving fuel cell efficiency. Tang *et al.* (2018)^10^ researched nanostructured materials and their electrochemical performance in improving the efficiency of solid oxide fuel cells. These studies underscore the crucial role of research in advancing fuel cell technology, inspiring further exploration and innovation in the field. Fuel cells consist of several key components, including the cathode, where electrochemical reactions occur in an oxygen or air atmosphere; the anode, which facilitates the absorption and reaction of hydrogen gas; the solid electrolyte, which enables ion or electron conduction; the interconnect, which connects individual cells; and the sealant, which prevents leaks and integrates the components. The cathode material plays a crucial role in fuel cell performance, requiring high electrical conductivity, oxidation resistance, chemical stability, dimensional stability under operational conditions, a thermal expansion coefficient compatible with other fuel cell components, and sufficient porosity to facilitate oxygen diffusion. Common cathode materials such as LaCoO_3_ and LaNiO_3_, oxides with a perovskite structure (general formula ABO_3_), are often enhanced through doping, a process of introducing impurities into a semiconductor to increase its electrical conductivity, with elements such as Sr, Co, and Fe to improve their electrical properties.^11,12^ Zhang *et al.* (2012)^13^ prepared double perovskite materials with the chemical formula Ba_2_MMoO_6_ (M = Fe, Co, Mn, Ni) as anode materials for solid oxide fuel cells. Their research demonstrated the high conductivity of Ba_2_MMoO_6_ compounds, particularly those with the highest performance, and highlighted their significant potential, achieving a maximum conductivity of 196 S cm^−1^ at 850 °C. This promising result paves the way for future solid oxide fuel cell technology advancements. Huang *et al.* (2018)^14^ synthesized PrBaCo_2−*x*_Mn_*x*_O_5+*δ*_ (*x* = 0, 0.5, 1) as cathode materials for intermediate-temperature solid oxide fuel cells. Their findings revealed a significant improvement in cathode performance with increasing Mn substitution in the Co site, marking a promising and exciting advancement in solid oxide fuel cell technology. Jin *et al.* (2018)^6^ prepared PrBaCo_2/3_Fe_2/3_Mn_1/3_O_5+*δ*_ (PBCFM2) using the sol–gel method as cathode materials for intermediate-temperature solid oxide fuel cells. PBCFM2, with its good thermal stability, not only exhibited potential and reassured its reliability for practical applications in the field. These findings open up new possibilities for developing solid oxide fuel cells that can operate at lower temperatures, potentially reducing costs and expanding the range of applications. Bannikov and Cherepanov (2006)^15^ studied LaNiO_3_ compounds synthesized *via* the citrate route and reported that LaNiO_3_ exhibited a single-phase structure after calcination at 800 °C, along with high electrical conductivity. These findings contribute to the understanding of LaNiO_3_ and suggest potential for further research in related applications. Carvalho *et al.* (2009)^8^ used the citrate method to synthesize La_*n*+1_Ni_*n*_O_3*n*+1−*δ*_ (*n* = 2, 3), resulting in the formation of oxygen vacancies within the structure. Zhang *et al.* (2010)^16^ synthesized LaSr_3_Fe_3_O_10−*δ*_ using the citrate acid route. Analysis revealed a tetragonal structure after calcination at 1200 °C. The material exhibited two conductivity regions: between 200–450 °C, where conductivity increased with temperature, and between 450–800 °C, where conductivity decreased as temperature rose. Basu *et al.* (2004)^3^ studied LaNi_1−*x*_Fe_*x*_O_3_ compounds and found that Fe substitution for Ni at *x* = 0.4 resulted in the highest electrical conductivity. Hou *et al.* (2014)^5^ investigated LaNi_0.6_Fe_0.4_O_3−*δ*_ compounds and found that Fe addition enhanced electrical conductivity. Basu *et al.* (2010)^17^ examined anodes using PtRu/C catalysts and cathodes with activated carbon. Glucose in the KOH solution served as the electrolyte, and cyclic voltammetry (CV) was used for analysis. The study reported a power density of 1.38 mW cm^−2^ and a current density of 2.74 mA cm^−2^ at a glucose concentration of 0.2 M in 1 M KOH. Cuevas-Muñiz *et al.* (2012)^18^ investigated Au/C as an anode catalyst and PtAg/C as a cathode catalyst with glucose as the electrolyte in alkaline fuel cells. The study observed a lower power density.

Among various synthesis techniques, high-energy ball milling has emerged as a promising method for the preparation of complex perovskite oxides due to its simplicity, scalability, and ability to induce solid-state reactions at lower temperatures.^19–21^ The high-energy collisions between balls and powder particles generate localized high temperatures and pressures, promoting atomic diffusion and homogeneous mixing at the nanoscale.

Compared to sol–gel or citrate methods, which typically require the use of solvents, complex precursors, and careful pH control, high-energy ball milling is a solvent-free, environmentally friendly process that avoids the formation of undesirable secondary phases often seen in wet-chemical routes.^22,23^ While sol–gel methods can offer better control over stoichiometry and lower calcination temperatures, they often involve longer preparation times and challenges in removing organic residues. Co-precipitation and spray pyrolysis, while useful for fine powders, may result in agglomeration and require complex post-treatment steps.

In contrast, high-energy ball milling offers significant advantages in terms of shorter synthesis time, enhanced reaction kinetics, and uniform particle size distribution, particularly beneficial for producing fine perovskite powders suitable for catalytic applications. Studies have shown that perovskite materials synthesized *via* high-energy ball milling exhibit improved phase purity, smaller crystallite size, and enhanced surface area, all of which are favorable characteristics for catalytic performance in fuel cell electrodes.^24^

This research aims to study the preparation and characterization of iron-doped lanthanum nickelate as a catalyst for air cathodes to enhance the performance of alkaline fuel cells. The lanthanum nickelate doped with iron is synthesized using high-energy ball milling through the solid-state reaction method. The study investigates the chemical composition, microstructure, and physical and electrical properties of the prepared ceramic materials, aiming to develop an efficient catalyst for air cathodes to improve fuel cell performance.

## Materials and methods

The preparation of lanthanum nickelate doped with iron (LaNi_(1−*x*)_Fe_*x*_O_3_; LNFO) begins with the selection of precursor materials: lanthanum oxide (La_2_O_3_), nickel oxide (NiO), and iron(iii) oxide (Fe_2_O_3_). The precursor powders are mixed in specific molar ratios of LaNi_(1−*x*)_Fe_*x*_O_3_, where *x* = 0.0, 0.2, 0.4, 0.6, 0.8, and 1.0. In the first step, 20 g of the appropriate mix is weighed out and then ground in an ethanol medium (25 ml) for 60 minutes using a high-energy ball mill to ensure a homogeneous mixture. After grinding, the sample is washed to remove excess ethanol and dried at approximately 150 °C on a hot plate with continuous stirring using a magnetic bar to ensure complete evaporation of solvents, followed by oven drying at 100 °C for 24 hours. Once dried, the powder is ground into a fine consistency using a mortar and pestle. The resulting powder is then transferred to an alumina crucible, sealed, and calcined at 800 °C for 2 hours using a heating rate of 5 °C min^−1^ to initiate the required solid-state reactions. This process facilitates the formation of the desired LaNi_(1−*x*)_Fe_*x*_O_3_ phase. After calcination, the powder undergoes a sintering process at 1000 °C for 2 hours to improve the structural integrity and densification of the material. The phase composition of the samples is evaluated through X-ray diffraction (XRD), which identifies the crystalline phases present. Additionally, thermal analysis techniques such as TGA (Thermogravimetric Analysis) and DSC (Differential Scanning Calorimetry) are employed to assess phase transitions or decomposition behavior. The microstructure is examined using scanning electron microscopy (SEM) and transmission electron microscopy (TEM) to observe the particle size and morphology, while energy dispersive X-ray spectroscopy (EDX) is utilized for elemental analysis. Finally, electrochemical properties, including the catalytic activity, are investigated through cyclic voltammetry (CV) measurements to assess the suitability of the materials for potential applications.

## Results and discussion

**Fig. 1:**
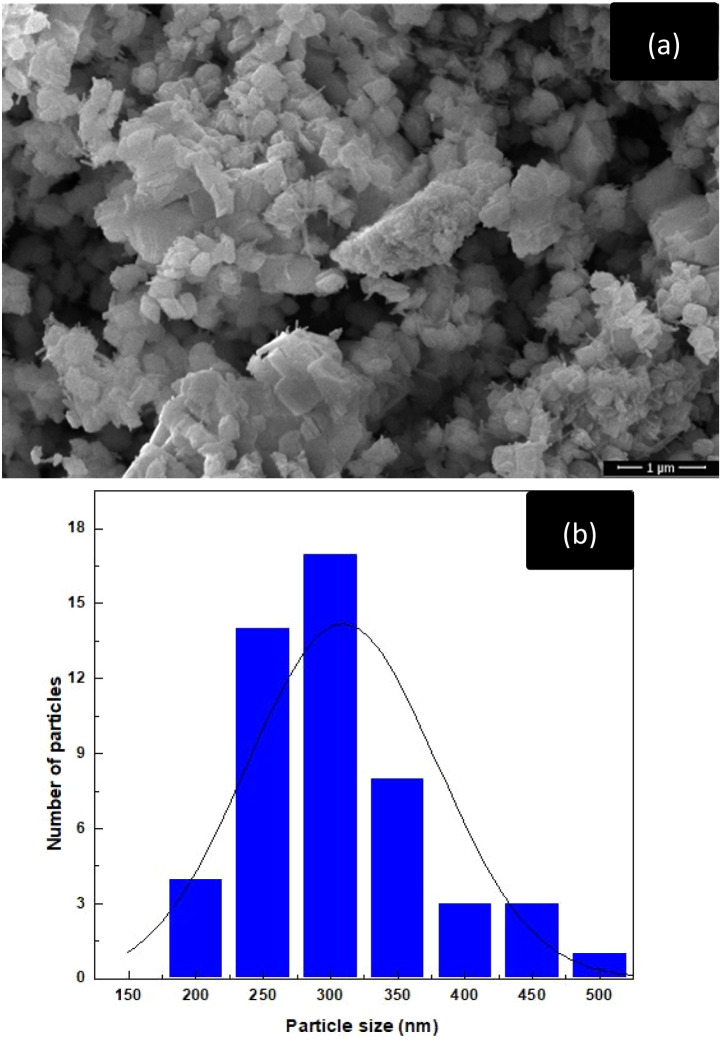
The precursor powders of La_2_O_3_, NiO, and Fe_2_O_3_: (a) SEM image, and (b) particle size distribution.

**Fig. 2:**
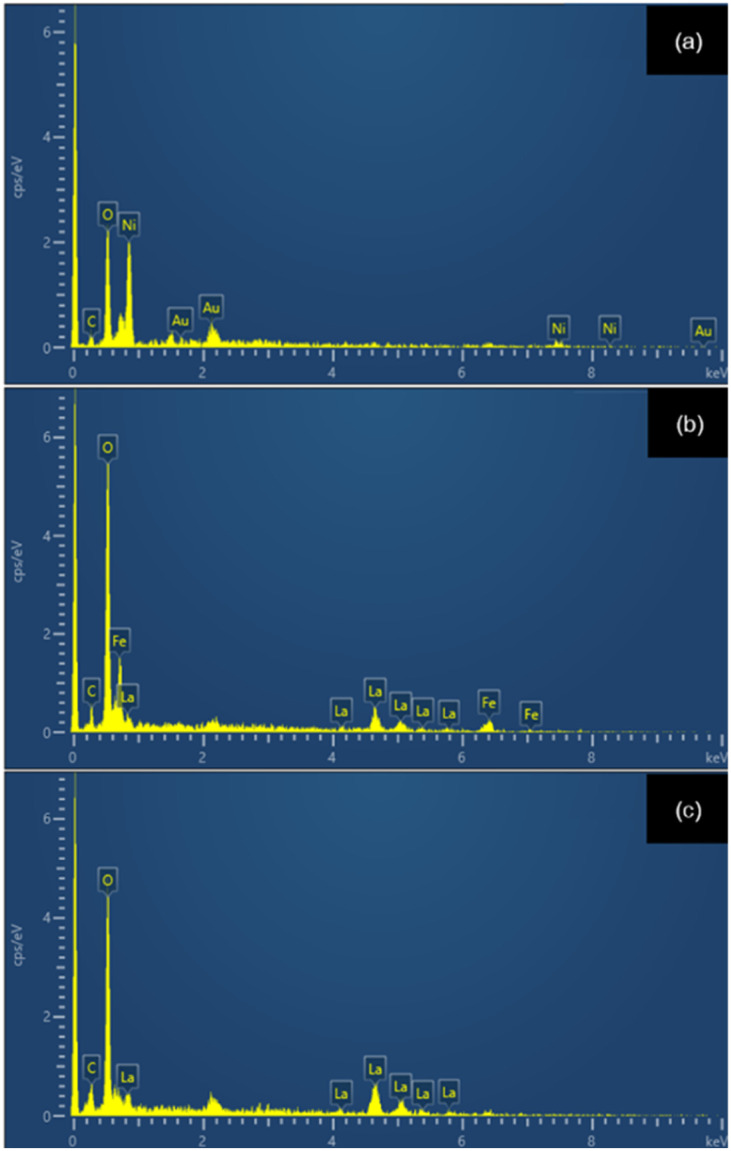
EDX spectra of La_2_O_3_, NiO, and Fe_2_O_3_: (a) region 1, (b) region 2, (c) region 3.

SEM analysis of La_2_O_3_, NiO, and Fe_2_O_3_ precursors at 500 00× magnification showed that the powders were made up of fine particles forming aggregated clusters with irregular polygonal shapes (Fig. 1a). The surfaces of the particles were rough and densely packed, suggesting that the precursor oxides consisted of primary particles assembled into agglomerates. Particle size analysis (Fig. 1b) revealed an average particle size of approximately 287 ± 53 nm. EDX confirmed the presence of La, Ni, Fe, and O, with minor C from carbon tape and gold from sample coating for conductivity (Fig. 2a–c). After the LNFO powder (LaNi_(1−*x*)_Fe_*x*_O_3_, where *x* = 0.0, 0.2, 0.4, 0.6, 0.8, and 1.0) underwent high-energy ball milling for 60 minutes, the powder was calcined at 800 °C. The microstructure was analyzed using a scanning electron microscope (SEM) with a magnification of 500 00×. The SEM images revealed that the LNFO particles had spherical, angular, and irregular shapes and formed agglomerates. These findings are consistent with the study by Vidal *et al.* (2015).^25^

**Fig. 3:**
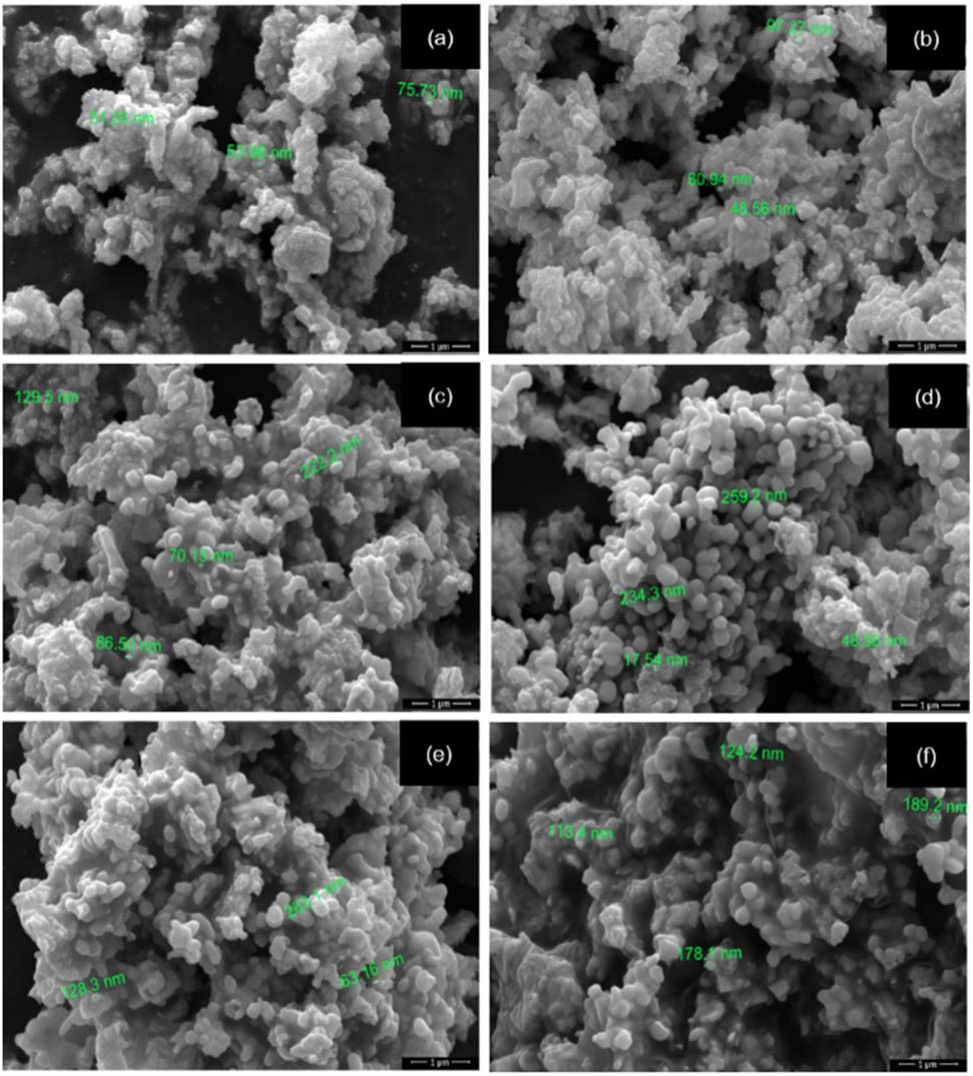
SEM images of LaNi_(1−*x*)_Fe_*x*_O_3_ calcined at 800 °C: (a) *x* = 0, (b) *x* = 0.2, (c) *x* = 0.4, (d) *x* = 0.6, (e) *x* = 0.8, and (f) *x* = 1.

As shown in Fig. 3a–f, the SEM micrographs of LaNi_1−*x*_Fe_*x*_O_3_ (*x* = 0–1) demonstrate a progressive increase in particle agglomeration and grain domain enlargement with higher Fe substitution. The undoped sample (*x* = 0) shows fine and uniformly dispersed particles, whereas the incorporation of Fe promotes stronger clustering and growth of the particle domains.

**Fig. 4:**
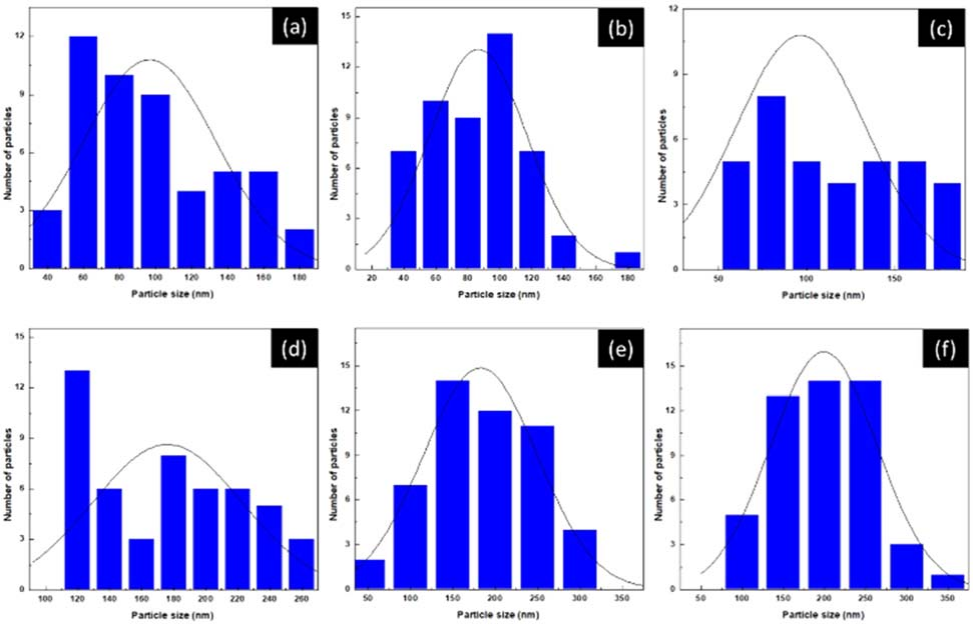
Particle size distribution of LaNi_(1−*x*)_Fe_*x*_O_3_ calcined at 800 °C: (a) *x* = 0, (b) *x* = 0.2, (c) *x* = 0.4, (d) *x* = 0.6, (e) *x* = 0.8, and (f) *x* = 1.

**Fig. 5:**
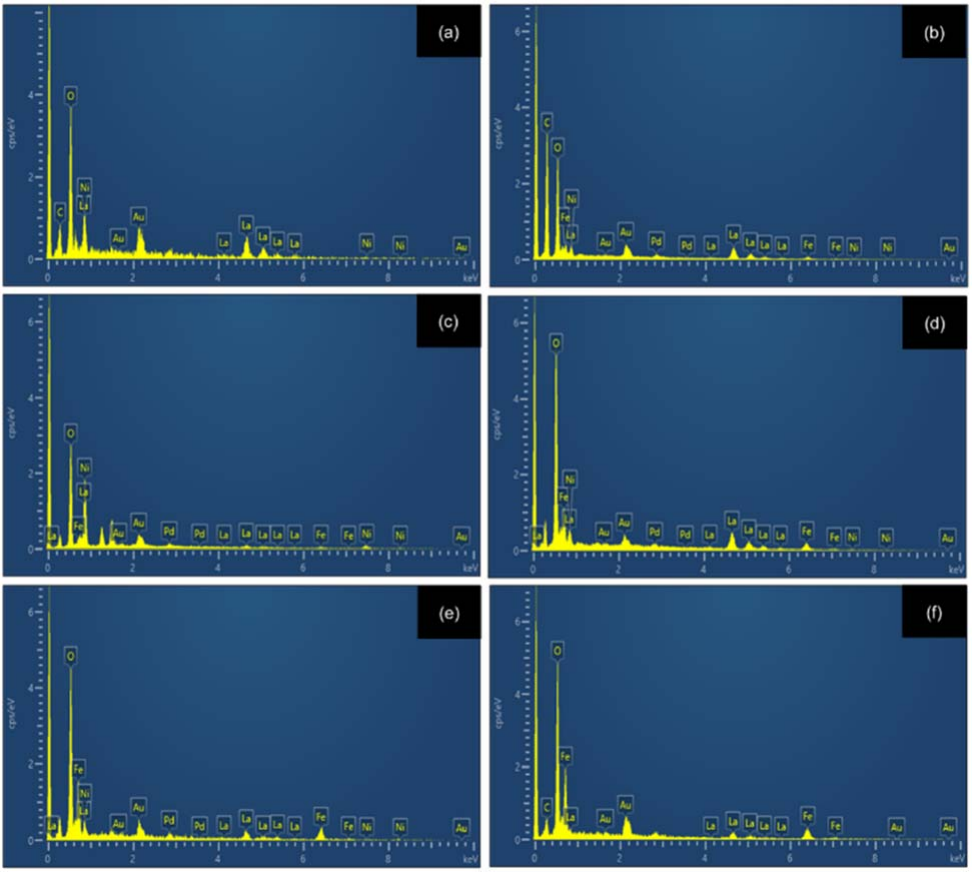
EDX spectra of LaNi_(1−*x*)_Fe_*x*_O_3_ powders calcined at 800 °C: (a) *x* = 0, (b) *x* = 0.2, (c) *x* = 0.4, (d) *x* = 0.6, (e) *x* = 0.8, and (f) *x* = 1.

This morphological evolution aligns well with the particle size distribution data presented in Fig. 4a–f, which exhibit a clear coarsening trend with increasing Fe content. For *x* = 0, most particles fall within 60–120 nm, while the distribution broadens to 70–150 nm at *x* = 0.2 and 90–160 nm at *x* = 0.4. More pronounced particle growth is observed at higher substitution levels, with sizes of 150–230 nm for *x* = 0.6 and 140–300 nm for *x* = 0.8. At *x* = 1, the particles reach their largest dimensions, predominantly within 160–330 nm, confirming the Fe-driven progressive coarsening behavior. Elemental analysis using energy-dispersive X-ray spectroscopy (EDX) confirmed the presence of La, Ni, Fe, and O, as shown in Fig. 5a–f. The LNFO powder (LaNi_(1−*x*)_Fe_*x*_O_3_, where *x* = 0.0, 0.2, 0.4, 0.6, 0.8, and 1.0) was sintered at 1000 °C for 2 hours with a 5 °C min^−1^ heating rate.

**Fig. 6:**
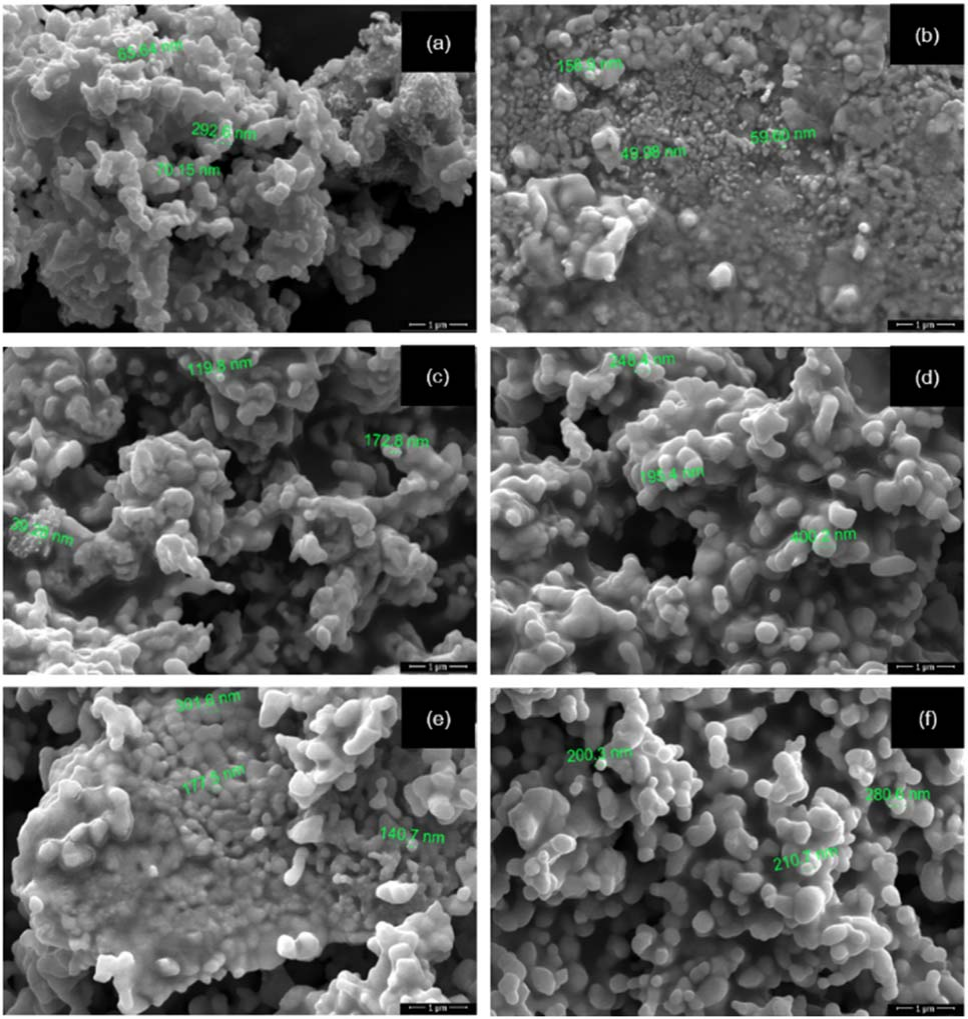
SEM images of LaNi_(1−*x*)_Fe_*x*_O_3_ powders calcined at 1000 °C: (a) *x* = 0, (b) *x* = 0.2, (c) *x* = 0.4, (d) *x* = 0.6, (e) *x* = 0.8, and (f) *x* = 1.

The microstructure was examined using a scanning electron microscope (SEM) at a magnification of 500 00×. For the sample with *x* = 0.0 (Fig. 6a–f), the grains appeared irregular and asymmetrical, with some showing incomplete growth. The grain sizes ranged from 49 to 500 nanometers. Neck formation between particles was observed, indicating that the particles were coming closer together, resulting in shrinkage and the formation of grain boundaries. These observations align with the findings of Vidal *et al.* (2015)^25^ and offer insights into the microstructure of LNFO powder.

**Fig. 7:**
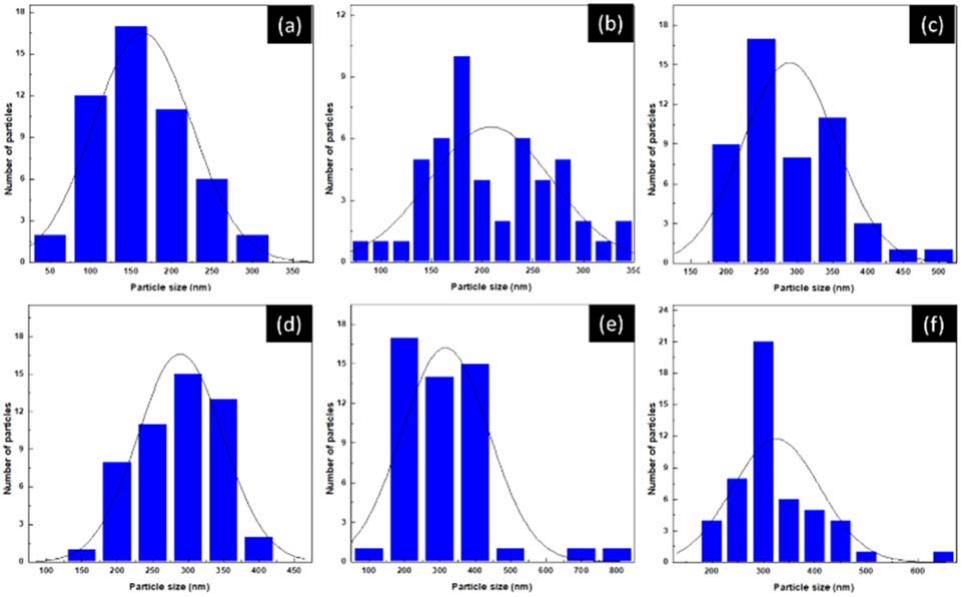
Particle size distribution of LaNi_(1−*x*)_Fe_*x*_O_3_ powders calcined at 1000 °C: (a) *x* = 0, (b) *x* = 0.2, (c) *x* = 0.4, (d) *x* = 0.6, (e) *x* = 0.8, and (f) *x* = 1.

**Fig. 8:**
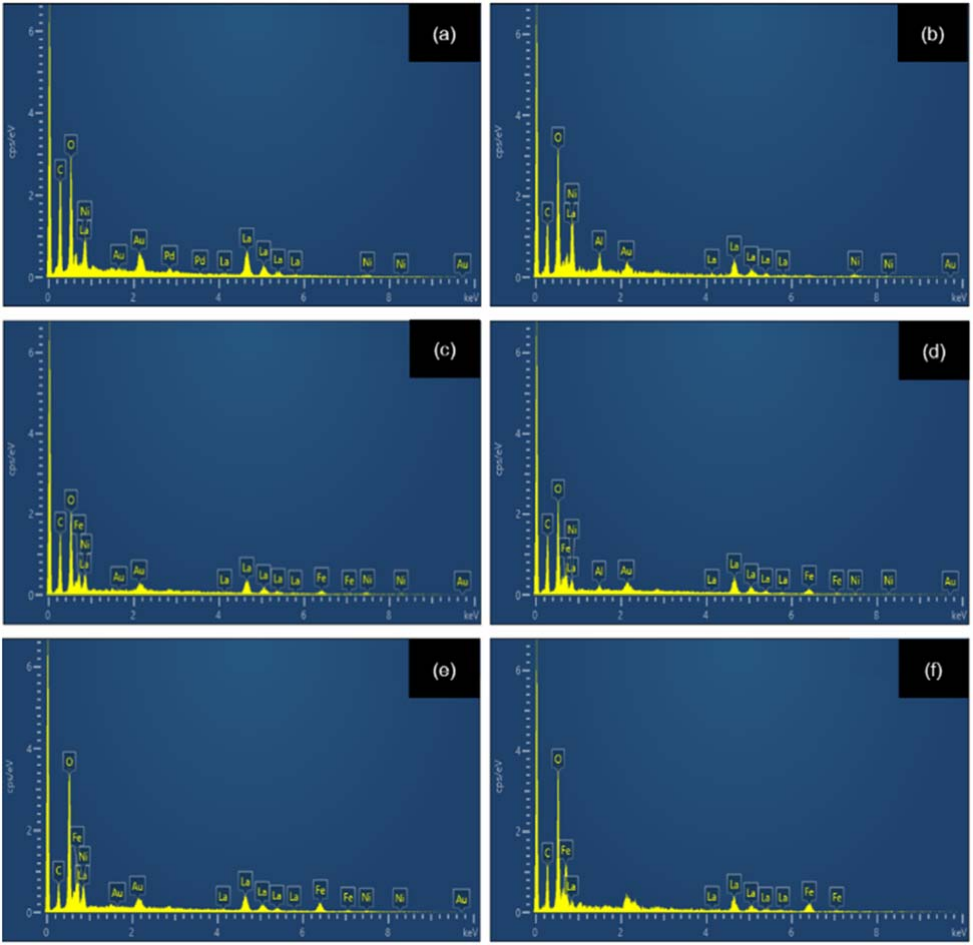
EDX spectra of LaNi_(1−*x*)_Fe_*x*_O_3_ powders calcined at 1000 °C: (a) *x* = 0, (b) *x* = 0.2, (c) *x* = 0.4, (d) *x* = 0.6, (e) *x* = 0.8, and (f) *x* = 1.

Fig. 7a–f shows the particle size distributions of LaNi_1−*x*_Fe_*x*_O_3_ powders calcined at 1000 °C. The average particle size increases systematically with Fe content. The undoped sample (*x* = 0) exhibits a narrow distribution centred at ∼150–200 nm, reflecting uniform grain growth. Introducing a small amount of Fe (*x* = 0.2–0.4) leads to broader, more asymmetric distributions, indicating the onset of defect-induced coalescence associated with lattice distortion and oxygen-vacancy formation. For *x* ≥ 0.6, the particle size further shifts toward larger values (250–600 nm), accompanied by a pronounced broadening of the distribution. The Fe-rich compositions (*x* = 0.8–1.0) show the largest particles, consistent with enhanced cation mobility and the high thermal stability of Fe-based perovskites. Overall, Fe substitution strongly modulates the grain-growth kinetics, driving a transition from uniform to defect-assisted coarsening. This compositional effect provides a useful handle for tuning microstructure and performance in La-based perovskites. Elemental analysis of the sintered LNFO powder at 1000 °C using energy-dispersive X-ray spectroscopy (EDX) confirmed the presence of La, Ni, Fe, and O, as shown in Fig. 8a–f.

**Fig. 9:**
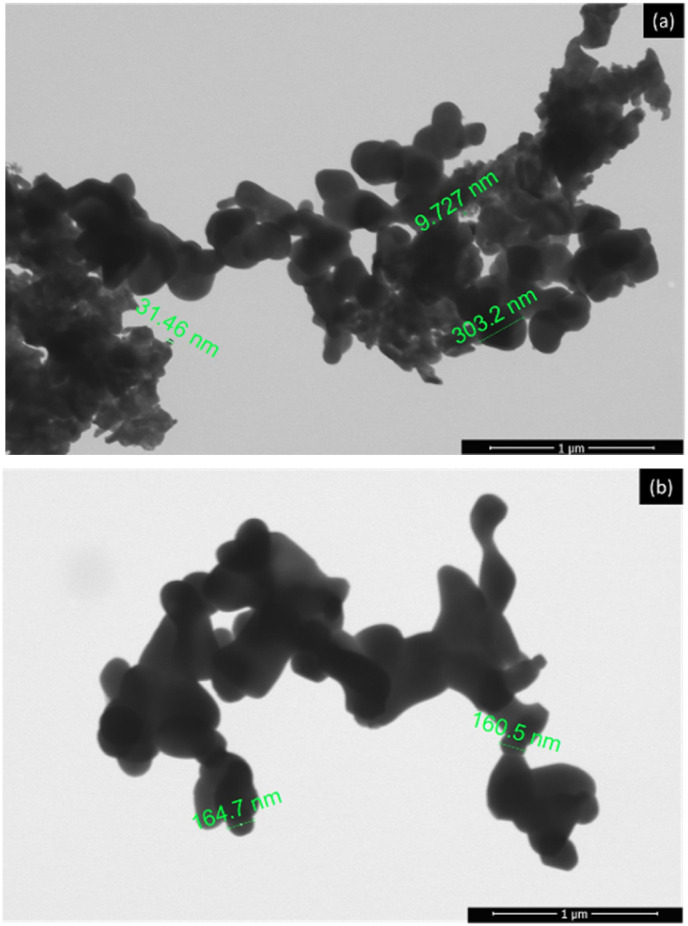
TEM images of LaNi_(1−*x*)_Fe_*x*_O_3_ (*x* = 0.4): (a) calcined at 800 °C and (b) sintered at 1000 °C.

The TEM analysis of LaNi_(1−*x*)_Fe_*x*_O_3_ powder with *x* = 0.4, calcined at 800 °C, as shown in Fig. 9a. The results revealed that the nanoparticle size of LaNi_0.6_Fe_0.4_O_3_ ranged from 50 to 400 nanometers. The nanoparticles exhibited various shapes, including spherical, angular, and irregular forms, and were clustered together with some single particles present. When the LaNi_0.6_Fe_0.4_O_3_ was sintered at 1000 °C, the TEM images shown in Fig. 9b demonstrated that the grain size had increased. This increase in grain size resulted from atomic diffusion, which caused the particles to come into closer contact, forming necks between adjacent particles. The grain shape became angular, and the grain size ranged from 100 to 600 nanometers.

**Fig. 10:**
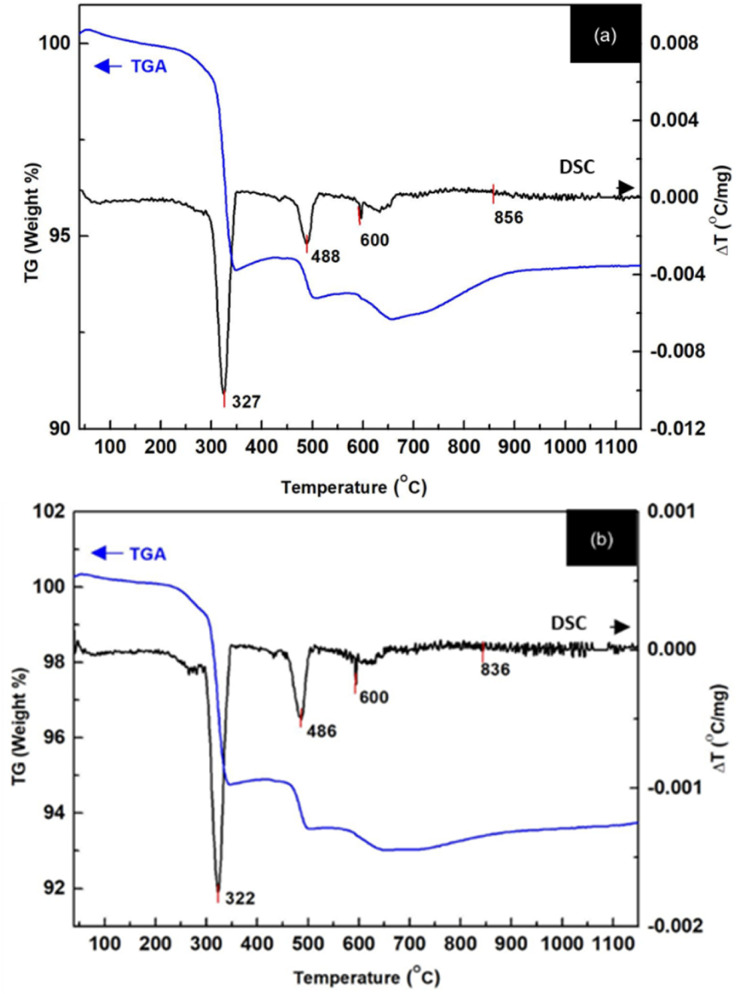
TGA-DSC curves of powders: (a) La_2_O_3_, and (b) LaNi_(0.6)_Fe_0.4_O_3_.

The thermal behavior of a La_2_O_3_ and NiO mixture was analyzed using Thermogravimetric Analysis (TGA) and Differential Scanning Calorimetry (DSC) by heating the sample from 30 °C to 1200 °C at a rate of 5 °C per minute (Fig. 10a and b). The TGA results revealed six stages of weight loss, each corresponding to specific chemical or structural changes within the material. At 162 °C, a small weight loss of 0.14% occurred, primarily due to the evaporation of water trapped in the crystalline structure of the La_2_O_3_ and NiO powder. This evaporation typically involves water adsorbed on the surface or within the material's pores. At around 245 °C, a weight loss of 0.77% was observed. This is likely due to the loss of volatile compounds or the decomposition of hydroxyl groups or oxygen-containing compounds in the mixture. At 310 °C, a more significant weight loss of 4.54% was noted, likely due to the decomposition of complex compounds. This involves the breakdown of oxygen–metal bonds, especially in the transition metal oxides, where oxygen may be released as metal oxides decompose. At 470 °C, a 1.17% weight loss occurred, which could be related to further decomposition of intermediate compounds. These changes likely involve the rearrangement of oxide phases. At 597 °C, a smaller weight loss of 0.55% was observed, which might be related to the continued release of oxygen or other volatile molecules from the oxides' crystalline structure. The DSC results confirmed these observations, showing phase transitions at specific temperatures. The first transition occurred at 323 °C, corresponding to the breakdown of water or weakly bound species. The second transition at around 486 °C suggests a more significant phase change, likely involving the rearrangement of oxide phases or the formation of new stable crystalline structures. The most substantial phase change occurred between 600 °C and 836 °C, marking a transformation in the crystal structure of the material, likely related to the decomposition or restructuring of the metal oxides.

**Fig. 11:**
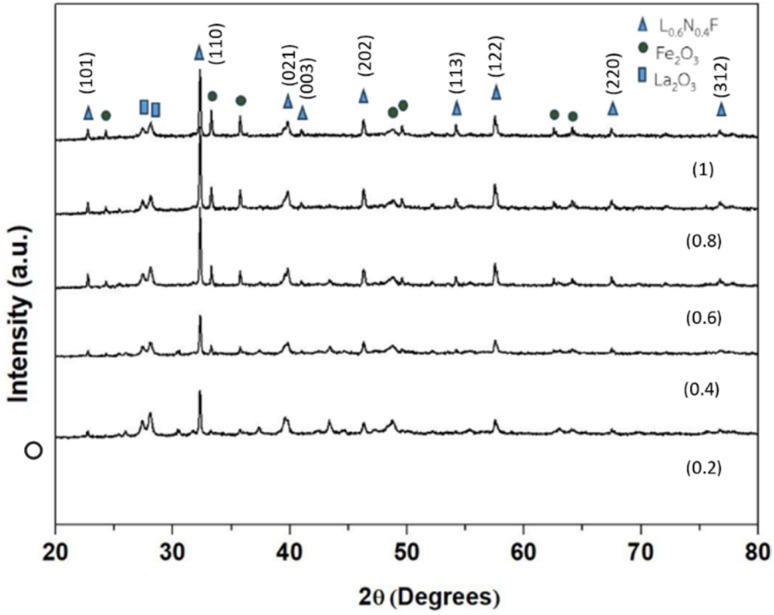
X-ray diffraction patterns of LaNi_(1−*x*)_Fe_*x*_O_3_ (*x* = 0.2, 0.4, 0.6 0.8 and 1) powder at 800 °C.

**Fig. 12:**
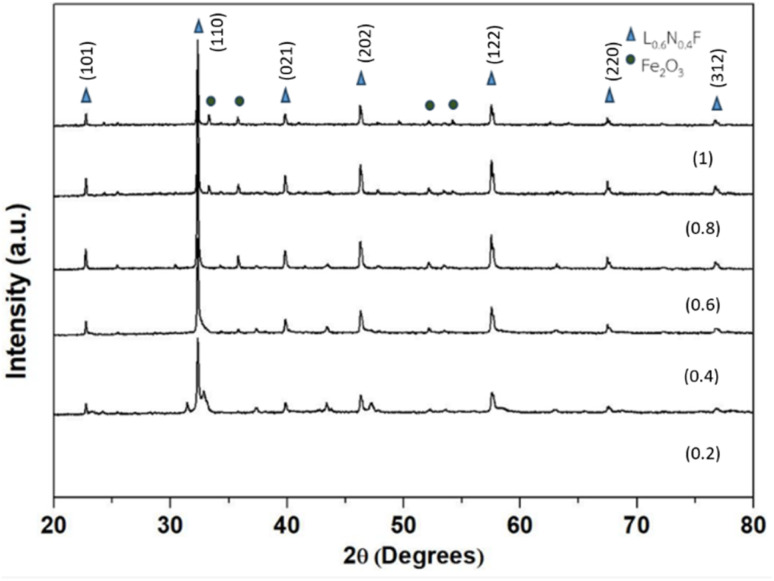
X-ray diffraction patterns of LaNi_(1−*x*)_Fe_*x*_O_3_ (*x* = 0.2, 0.4, 0.6 0.8 and 1) ceramics at 1000 °C.

The LaNi_(1−*x*)_Fe_*x*_O_3_ powders with *x* values of 0.2, 0.4, 0.6, 0.8, and 1 were calcined at 800 °C for 2 hours with a heating rate of 5 °C min^−1^ and then sintered at 1000 °C. The crystal phase of these samples was analyzed using X-ray diffraction (XRD) in the 2*θ* range of 20° to 80° and compared with standard data from the JCPDS database. The XRD patterns of the samples calcined at 800 °C showed that all of them exhibited a rhombohedral crystal structure of LaNiO_3_, consistent with the JCPDS file no. 33-0711.^26,27^ However, the LaNiO_3_ phase was incomplete and impure, with the presence of the La_2_O_3_ phase, which appeared at 2*θ* = 27°, matching the JCPDS file no. 03-065-3185. The Fe_2_O_3_ phase was also observed, corresponding to JCPDS file no. 01-089-0598, as shown in Fig. 11. After sintering at 1000 °C, the XRD results again confirmed the presence of the LaNiO_3_ rhombohedral phase, consistent with JCPDS file no. 33-0711, along with the Fe_2_O_3_ phase, as shown in Fig. 12. These findings align with the results reported by Mahmoud *et al.* (2015)^28^ and Basu *et al.* (2004).^3^

**Fig. 13:**
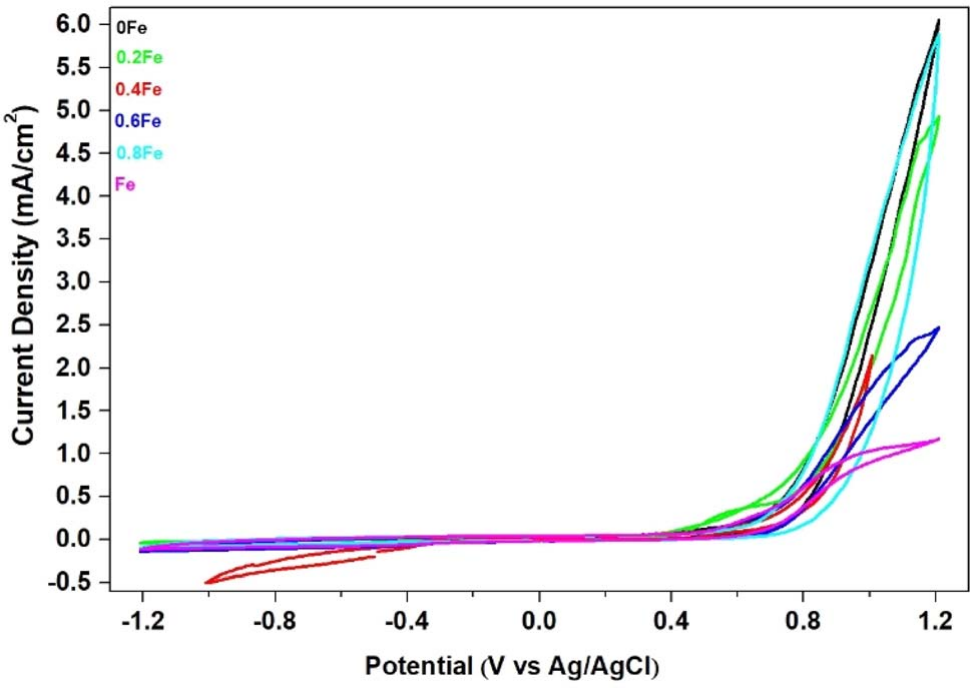
CV curves of LaNi_(1−*x*)_Fe_*x*_O_3_ (*x* = 0, 0.2, 0.4, 0.6 0.8 and 1).

The electrical properties of the perovskite LaNi_(1−*x*)_Fe_*x*_O_3_ were studied to evaluate its electrochemical characteristics as a catalyst for fuel cells. The catalyst was tested as a cathode in alkaline fuel cells to improve performance by promoting the reduction reaction while minimizing the oxidation reaction at the cathode. Electrochemical behavior was assessed using Cyclic Voltammetry (CV), focusing on its performance in a KOH solution with sorbitol. The result shows that the cyclic voltammogram of the LNFO/C (LaNi_(1−*x*)_Fe_*x*_O_3_ where *x* = 0.0, 0.2, 0.6, 0.8, and 1.0) catalyst showed no clear oxidation peak due to sorbitol adsorption on the catalyst metal. However, an oxidation peak was observed for LaNi_(1−*x*)_Fe_*x*_O_3_ with *x* = 0.4 due to sorbitol adsorption on the catalyst metal, as shown in Fig. 13. The cyclic voltammogram of LaNi_0_._6_Fe_0_._4_O_3_ measured within −1.0 to +1.1 V *vs.* Ag/AgCl exhibits well-defined redox behavior arising from the transition-metal cations embedded in the perovskite lattice. During the cathodic sweep, the current decreases progressively from around −0.1 V and reaches a minimum near −0.5 to −0.55 mA cm^−2^, which is attributed to the overlap of the Ni^3+^/Ni^2+^ and Fe^3+^/Fe^2+^ reduction processes. The broad nature of this reduction feature reflects the distributed and surface-mediated electron transfer typical of mixed-oxide perovskites with heterogeneous catalytic sites. Upon the anodic reversal, the current increases smoothly across 0–0.4 V, corresponding to the re-oxidation of Ni and Fe species, and then rises sharply beyond 0.6–0.7 V, reaching nearly 1.0 mA cm^−2^ near +1.0 V. This steep increase signifies the onset of the oxygen evolution reaction (OER), demonstrating that Fe-doped LaNiO_3_ becomes highly electrocatalytically active at relatively low overpotentials. The moderate hysteresis between the forward and reverse scans suggests partial pseudocapacitive behavior originating from surface adsorption–desorption processes, while also indicating reasonable reversibility of the Ni/Fe redox couple. These electrochemical signatures show that Fe incorporation at *x* = 0.4 not only improves the redox accessibility but also enhances the intrinsic OER activity of the perovskite. This behavior is consistent with previous reports in which Fe has been shown to significantly activate the OER performance of LaNiO_3_-based catalysts through surface reconstruction and formation of mixed Ni–Fe oxyhydroxide phases.^29,30^ Furthermore, the performance trends observed here align with the broader understanding of Fe-containing perovskites as highly efficient alkaline OER catalysts, as demonstrated in recent perovskite catalyst studies.^31,32^ Collectively, the CV results confirm that LaNi(1−*x*)FexO_3_ (*x* = 0.4) possesses strong bifunctional redox characteristics, supporting its suitability as a promising air-cathode catalyst in alkaline fuel cell systems.

## Conclusions

The preparation of powder and ceramics of lanthanum nickelate doped with iron (LaNi_(1−*x*)_Fe_*x*_O_3_, where *x* = 0.0, 0.2, 0.4, 0.6, 0.8, and 1.0) was carried out using an oxide mixing method through high-energy ball milling, followed by calcination at 800 °C and sintering at 1000 °C. The physical and electrical properties were investigated, and the results show that the precursor materials La_2_O_3_, NiO, and Fe_2_O_3_ were analyzed before the high-energy milling process using scanning electron microscopy (SEM). The LNFO precursor's particle size was 300–1000 nm, with a characteristic angular shape. Energy dispersive X-ray spectroscopy (EDX) analysis confirmed that the material consisted of La, Ni, Fe, and O elements. The LNFO powder (LaNi_(1−*x*)_Fe_*x*_O_3_, where *x* = 0.0, 0.2, 0.4, 0.6, 0.8, and 1.0) was subjected to high-energy ball milling for 60 minutes and calcined at 800 °C. The results showed that the LNFO had a spherical, angular, and irregular shape, with agglomerated particles in the 30–400 nm range. After sintering at 1000 °C, the grain morphology appeared irregularly angular, with grain sizes ranging from 50 to 600 nm. TEM analysis of LaNi_(1−*x*)_Fe_*x*_O_3_ (*x* = 0.4) powder calcined at 800 °C showed a particle size in the 50–400 nm range. After sintering at 1000 °C, the particle size increased to 100–600 nm.

Thermogravimetric analysis of La_2_O_3_ and NiO powder revealed phase transformation between 600–836 °C. The crystallization of LNFO was confirmed through X-ray diffraction analysis, which showed that the LaNi_(1−*x*)_Fe_*x*_O_3_ structure at *x* = 0.2, 0.4, 0.6, 0.8, and 1.0 exhibited a rhombohedral structure after sintering at 1000 °C. The electrochemical characteristics of the LNFO catalyst were studied using cyclic voltammetry. The results indicated that the CV of the LNFO/C catalyst (LaNi_(1−*x*)_Fe_*x*_O_3_, with *x* = 0.4) in a 0.1 M KOH alkaline solution, with 0.1 M ethanol, showed oxidation peaks due to ethanol adsorption on the catalyst metal, confirming the electrochemical behavior of the catalyst.

## Conflicts of interest

There are no conflicts to declare.

## Data Availability

The original data supporting the findings of this study are presented within the article. Further inquiries can be made by contacting the corresponding author at schontira@tsu.ac.th.
